# Thyroid autoimmunity does not delineate a cardiometabolic or androgenic phenotype in women with polycystic ovary syndrome: a pre-specified cross-sectional analysis

**DOI:** 10.3389/fendo.2026.1839476

**Published:** 2026-05-01

**Authors:** Natalia Piórkowska, Lech Madeyski, Marcin Leśniewski, Grzegorz Franik, Anna Bizoń

**Affiliations:** 1 Faculty of Information and Communication Technology, Wroclaw University of Science and Technology, Wrocław, Poland; 2 Department of Medical Anthropology, Faculty of Medical Sciences, Medical University of Silesia, Katowice, Poland; 3 Department of Endocrinological Gynecology, Faculty of Medical Sciences, Medical University of Silesia, Katowice, Poland; 4 Department of Toxicology, Faculty of Pharmacy, Wroclaw Medical University, Wrocław, Poland

**Keywords:** androgens, cardiometabolic risk, insulin resistance, oral glucose tolerance test, polycystic ovary syndrome, thyroid autoimmunity, triglyceride-to-HDL cholesterol ratio

## Abstract

**Background:**

Thyroid autoimmunity (TAI) is frequently reported in women with polycystic ovary syndrome (PCOS), yet its clinical relevance for cardiometabolic and androgenic severity remains uncertain. We evaluated whether TAI identifies a metabolically or androgenically more severe PCOS phenotype using pre-specified exposure definitions and cardiometabolic endpoints.

**Methods:**

This cross-sectional study included 1,300 women with confirmed PCOS in the source dataset. Thyroid autoimmunity was defined *a priori* using three definitions: anti-thyroid peroxidase antibodies above the laboratory upper limit of normal (TAI_A, primary definition), anti-TPO positivity combined with thyroid-stimulating hormone >4.0 mIU/L (TAI_B), and high-titer anti-TPO >100 IU/mL (TAI_C). The primary endpoint was triglyceride-to-high-density lipoprotein cholesterol ratio (TG/HDL-C) >3.5. Secondary endpoints included non-HDL-C ≥130 mg/dL and 120-minute oral glucose tolerance test (OGTT) glucose ≥140 mg/dL. Associations were assessed using age-adjusted Firth logistic regression models in complete-case cohorts. Sensitivity analyzes included restriction to euthyroid participants, alternative TAI definitions, trimming of extreme values (1–99%), and bootstrap-based confidence intervals. Exploratory hormonal comparisons were adjusted using the Benjamini–Hochberg false discovery rate.

**Results:**

TAI_A was not significantly associated with the primary endpoint (TG/HDL >3.5) (OR 0.77, 95% CI 0.21–1.67). No significant associations were observed for secondary endpoints including non-HDL-C ≥130 mg/dL (OR 1.09, 95% CI 0.61–1.76) or impaired glucose tolerance on OGTT (OR 1.27, 95% CI 0.63–2.18). Results remained directionally consistent across alternative TAI definitions and sensitivity analyzes, including restriction to euthyroid women and trimming of extreme values. In exploratory analyzes, thyroid-stimulating hormone levels differed between TAI-positive and TAI-negative women, while no androgenic or cardiometabolic parameters remained significant after false discovery rate correction. Model diagnostics did not indicate major violations of model assumptions.

**Conclusion:**

In this large cross-sectional cohort of women with PCOS, thyroid autoimmunity was not associated with an adverse cardiometabolic or androgenic phenotype. Anti-TPO positivity alone therefore does not appear to identify a metabolically high-risk PCOS subgroup under the studied conditions. Prospective studies are needed to clarify the longitudinal implications of thyroid autoimmunity in PCOS.

## 1 Introduction

Polycystic ovary syndrome (PCOS) is the most common endocrine disorder in women of reproductive age and is characterized by hyperandrogenism, ovulatory dysfunction, and polycystic ovarian morphology (1). Beyond reproductive features, PCOS is increasingly recognized as a heterogeneous metabolic condition associated with insulin resistance, dyslipidemia, and increased cardiometabolic risk (2). Considerable effort has therefore been directed toward identifying clinically meaningful PCOS subphenotypes that may differ in metabolic severity and long-term risk profiles (3, 4).

Thyroid autoimmunity (TAI), most commonly defined by the presence of anti–thyroid peroxidase antibodies (anti-TPO), has been reported to occur more frequently in women with PCOS compared with the general population (5, 6). Autoimmune thyroid disease has been reported in 18–40% of PCOS women, depending on PCOS diagnostic criteria and ethnicity (6). This co-occurrence has raised interest in potential shared mechanisms, including chronic low-grade inflammation, immune dysregulation, and interactions between metabolic and endocrine pathways (7–9). The concept of an immune–metabolic axis has gained traction in recent years, suggesting that autoimmune activity could contribute to metabolic dysfunction through inflammatory signaling, alterations in insulin sensitivity, or modulation of steroidogenesis. Within this framework, it has been hypothesized that thyroid autoimmunity might identify a subset of women with PCOS characterized by greater metabolic or androgenic severity.

However, the clinical implications of thyroid autoimmunity in PCOS remain unclear. Previous studies have produced inconsistent findings, with some reports suggesting associations between TAI and adverse lipid profiles, insulin resistance, or impaired glucose tolerance, while others have failed to demonstrate such relationships (9, 10). Many of these studies were limited by small sample sizes, heterogeneous definitions of thyroid autoimmunity, varying thyroid functional states, or insufficient adjustment strategies. Furthermore, few investigations have evaluated cardiometabolic endpoints using clearly pre-specified thresholds or conducted robustness analyzes across alternative definitions of TAI. As a result, whether thyroid autoimmunity meaningfully delineates a metabolically or androgenically more severe PCOS phenotype remains unresolved.

The present study aimed to address this gap using a pre-specified cross-sectional design in a large cohort of women with PCOS. Thyroid autoimmunity was defined *a priori* using three operational definitions, including anti-TPO positivity above the laboratory upper limit of normal and a high-titer threshold. The primary endpoint was an elevated triglyceride-to-high-density lipoprotein cholesterol ratio (TG/HDL-C >3.5), selected as a clinically relevant marker of atherogenic risk and insulin resistance. Secondary endpoints included non–HDL-C ≥130 mg/dL and impaired glucose tolerance defined by 120-minute oral glucose tolerance test (OGTT) glucose ≥140 mg/dL. We hypothesized that thyroid autoimmunity would be associated with a more adverse cardiometabolic and androgenic profile. To ensure robustness, analyzes incorporated predefined sensitivity strategies and model diagnostics.

## 2 Materials and methods

### 2.1 Study design and population

This study was a pre-specified cross-sectional analysis of women with PCOS evaluated at a tertiary endocrine referral center. The analysis used an existing clinical dataset comprising women who underwent standardized clinical, biochemical, and metabolic assessment as part of routine care.

PCOS was diagnosed according to the Rotterdam criteria, requiring the presence of at least two of the following features after exclusion of other etiologies: (1) oligo- or anovulation, (2) clinical or biochemical hyperandrogenism, and (3) polycystic ovarian morphology on ultrasound.

The source dataset included 1,300 women with confirmed PCOS. Participants were eligible for the present analysis if measurements of anti-TPO were available to define thyroid autoimmunity status. Women with missing anti-TPO measurements were excluded from analyzes requiring TAI classification.

For each endpoint analysis, complete-case cohorts were defined based on the availability of variables required to construct the corresponding outcome and covariates. Consequently, the number of analyzed participants varied across endpoints. The selection process from the source dataset to endpoint-specific analytic cohorts is illustrated in the STROBE participant flow diagram.

The study was conducted in accordance with the principles of the Declaration of Helsinki. Ethical approval was obtained from the institutional review board of the participating center (approval number: to be inserted). As this study involved secondary analysis of routinely collected clinical data, informed consent procedures followed local regulatory requirements.

### 2.2 Clinical and laboratory assessment

All participants underwent standardized clinical evaluation and fasting venous blood sampling as part of routine clinical assessment at the study center.

Laboratory measurements included the following domains:

#### 2.2.1 Thyroid and androgen-related parameters

Serum thyroid-stimulating hormone (TSH), free thyroxine (FT4), anti-TPO, and steroid hormones (including total testosterone (TT), free testosterone (FT), dehydroepiandrosterone sulfate (DHEA-S) were measured using electrochemiluminescence immunoassays (ECLIA) on a cobas^®^ pro integrated solution analyzer (Roche Diagnostics, Mannheim, Germany), a fully automated platform for combined clinical chemistry and immunoassay testing.

#### 2.2.2 Lipid profile

Serum lipid parameters, including total cholesterol (TC), high-density lipoprotein cholesterol (HDL-C), low-density lipoprotein cholesterol (LDL-C), and triglycerides (TG), were determined using enzymatic colorimetric methods with commercially available reagents (Roche Diagnostics) on an automated analyzer.

Derived lipid indices were calculated as follows: non–HDL cholesterol (non–HDL-C) was defined as TC minus HDL-C, and the triglyceride-to-HDL cholesterol ratio (TG/HDL-C) was calculated as TG divided by HDL-C.

#### 2.2.3 Glucose metabolism

Plasma glucose concentrations were measured using an enzymatic colorimetric method (Roche Diagnostics). All participants underwent a standard 75 g oral glucose tolerance test (OGTT).

All biochemical analyzes were performed in the same certified clinical laboratory using standardized assays implemented in routine clinical practice, at the Central Laboratory of the University Clinical Centre of the Medical University of Silesia in Katowice, Poland.

### 2.3 Definition of thyroid autoimmunity

Thyroid autoimmunity (TAI) was defined *a priori* using three pre-specified definitions based on anti-TPO and TSH levels.

#### 2.3.1 Primary definition (TAI_A)

The primary exposure definition (TAI_A) was defined as anti-TPO concentration above the upper limit of normal (ULN) according to the reference range of the local laboratory performing the assays.

#### 2.3.2 Alternative definitions

Two additional definitions of thyroid autoimmunity were evaluated in sensitivity analyzes:

- TAI_B: anti-TPO above ULN combined with elevated TSH (>4.0 mIU/L), representing autoimmune thyroid disease with biochemical thyroid dysfunction.
- TAI_C: high-titer anti-TPO positivity (anti-TPO >100 IU/mL), representing a more stringent definition of thyroid autoimmunity.

All primary analyzes used TAI_A as the main exposure variable, whereas TAI_B and TAI_C were examined in sensitivity analyzes to evaluate the robustness of associations across alternative definitions of thyroid autoimmunity.

### 2.4 Cardiometabolic and androgenic endpoints

#### 2.4.1 Primary endpoint

The pre-specified primary endpoint was an elevated TG/HDL-C ratio, defined as TG/HDL-C >3.5. This threshold has been widely used as a surrogate marker of atherogenic dyslipidemia and insulin resistance in cardiometabolic risk studies (11–13). Cut-off values ranging from 3.0 to 3.5 have been proposed; therefore, a threshold of 3.5 was selected as a conservative indicator of increased cardiometabolic risk.

#### 2.4.2 Secondary endpoints

Secondary cardiometabolic endpoints included:

- non–HDL-C ≥130 mg/dL, and
- 120-minute plasma glucose ≥140 mg/dL during the OGTT.

The non-HDL-C threshold of ≥ 130 mg/dL was selected based on established lipid management guidelines as a clinically relevant marker of atherogenic lipoprotein burden and cardiovascular risk (14, 15).

A 120-minute plasma glucose ≥140 mg/dL during OGTT corresponds to impaired glucose tolerance according to WHO and ADA diagnostic criteria and reflects early disturbances in glucose metabolism associated with increased cardiometabolic risk (16, 17).

All cardiometabolic endpoints were analyzed as binary outcomes in regression models.

#### 2.4.3 Androgenic parameters

Androgen-related parameters (TT, FT, and DHEAS) were analyzed as continuous variables in exploratory comparisons between TAI-positive and TAI-negative participants. To account for multiple testing, p-values from these exploratory analyzes were adjusted using the Benjamini–Hochberg false discovery rate (FDR) procedure.

### 2.5 Data processing and quality control

All data processing procedures were pre-specified and implemented using reproducible analysis scripts.

#### 2.5.1 Units and harmonization

Laboratory values were reviewed for consistency of measurement units. When necessary, unit harmonization procedures were applied to ensure comparability across measurements.

#### 2.5.2 Parsing and numeric cleaning

Laboratory entries recorded as ranges (e.g., “a–b”) were converted to numeric values using predefined parsing rules. Entries reported as threshold values (e.g., “<x” or “>x”) were conservatively mapped to the corresponding boundary value to preserve numerical interpretability.

#### 2.5.3 Derived variables

The cardiometabolic indices TG/HDL-C and non–HDL-C were calculated as described above.

#### 2.5.4 Transformation and trimming

Variables with right-skewed distributions (e.g., anti-TPO or TG) were evaluated using log-transformation for descriptive and visualization purposes. In sensitivity analyzes, extreme values were trimmed at the 1st and 99th percentiles (1–99% trimming) to assess the robustness of regression estimates.

#### 2.5.5 Missing data

Patterns of missingness were evaluated descriptively. Primary analyzes were conducted using complete-case data for each endpoint-specific model, and the number of analyzed observations therefore varied depending on variable availability. No imputation procedures were performed.

#### 2.5.6 Outlier and influence assessment

Potentially influential observations were evaluated using standardized residuals and Cook’s distance diagnostics within regression models. These diagnostics were used to assess model stability rather than to automatically exclude observations.

### 2.6 Statistical analysis

All statistical analyzes were performed using Python (version 3.12) with standard scientific libraries (NumPy, pandas, SciPy, statsmodels, and scikit-learn). All tests were two-sided, and p-values <0.05 were considered statistically significant unless otherwise specified.

#### 2.6.1 Primary analysis

The association between thyroid autoimmunity (TAI_A) and the primary endpoint (TG/HDL-C >3.5) was assessed using Firth logistic regression, which reduces small-sample bias in logistic models.

Models were adjusted for age as a minimal *a priori* confounder. Effect estimates are reported as odds ratios (ORs) with 95% confidence intervals (CIs). Confidence intervals were obtained using bootstrap resampling to provide robust interval estimation.

#### 2.6.2 Secondary analyzes

Separate regression models were fitted for the secondary cardiometabolic endpoints (non–HDL-C ≥130 mg/dL and OGTT 120-minute glucose ≥140 mg/dL), using the same modeling strategy and age adjustment as in the primary analysis.

Selected metabolic and hormonal parameters were additionally evaluated in exploratory continuous analyzes comparing TAI-positive and TAI-negative groups.

#### 2.6.3 Sensitivity analyzes

Robustness of the primary findings was assessed using multiple pre-specified sensitivity analyzes:

- Restriction to euthyroid participants (TSH within the reference range and normal FT4).
- Use of alternative TAI definitions (TAI_B and TAI_C).
- Trimming of extreme values (1st–99th percentiles).
- Bootstrap-based confidence interval estimation.

#### 2.6.4 Multiple testing control

For exploratory comparisons involving multiple hormonal and metabolic parameters, p-values were adjusted using the Benjamini–Hochberg false discovery rate (FDR) procedure.

#### 2.6.5 Model diagnostics

Model calibration was evaluated using calibration plots and the Hosmer–Lemeshow goodness-of-fit test.

Potentially influential observations were examined using Cook’s distance diagnostics.

Multicollinearity among predictors was assessed using variance inflation factors (VIFs).

The assumption of linearity for the age covariate was evaluated using spline-based sensitivity models.

Because some standard diagnostic procedures are not directly available for Firth logistic regression, selected diagnostic measures were computed using auxiliary standard logistic models fitted to the same complete-case cohorts.

## 3 Results

### 3.1 Study population characteristics

A total of 1,300 women with confirmed PCOS were included in the source dataset. Measurements of anti-TPO were available for 1,055 participants, allowing classification of thyroid autoimmunity status. Among these, 84 women (8.0%) were classified as TAI-positive according to the primary definition (anti-TPO above the laboratory upper limit of normal), whereas 971 women (92.0%) were classified as TAI-negative.

Baseline clinical and biochemical characteristics stratified by TAI status are summarized in **Table 1**. Women with TAI showed higher TSH concentrations, consistent with the known physiological relationship between thyroid autoimmunity and thyroid function.

**Table 1:** Baseline characteristics.

Variable	TAI-positive (n=84)	TAI-negative (n=971)	Missing, n	SMD	P value
Clinical characteristics					
Age (years)	22 [21, 23]	22 [20, 23]	0	0.103	1.000
Thyroid parameters					
TSH (mIU/L)	2.4 [1.46, 3.52]	1.77 [1.27, 2.42]	1	0.896	<0.001
Anti-TPO (IU/mL)	129 [70.9, 261]	9.9 [9, 13]	0	4.247	<0.001
Lipid profile					
TG (mg/dL)	75.3 [58.8, 103]	77 [58.9, 105]	2	-0.018	0.718
HDL-C (mg/dL)	56.6 [48, 66]	57.8 [48.6, 67.2]	2	-0.012	0.497
TC (mg/dL)	164 [137, 188]	164 [146, 185]	2	-0.048	0.948
LDL-C (mg/dL)	85.5 [68.7, 107]	87.9 [72, 106]	2	-0.043	0.560
Derived lipid indices					
TG/HDL-C ratio	1.24 [0.95, 2.22]	1.29 [0.904, 2]	2	0.048	0.669
Non-HDL-C (mg/dL)	103 [81.9, 127]	104 [86.8, 125]	2	-0.043	0.677
Glucose metabolism					
Glu0 (mg/dL)	84 [79.9, 89.4]	83.7 [80.3, 87.9]	19	0.075	0.831
OGlu120 (mg/dL)	106 [93.2, 126]	108 [91.2, 127]	20	0.031	0.693
Androgen-related parameters					
TT (ng/mL)	0.369 [0.258, 0.483]	0.37 [0.272, 0.517]	1	-0.128	0.957
FT (pg/mL)	2.71 [1.41, 3.94]	2.14 [1.19, 3.65]	118	0.074	0.027
DHEAS (µg/mL)	316 [245, 419]	320 [245, 413]	1	-0.094	0.814
Androstenedione (ng/mL)	1.42 [1.12, 1.76]	1.48 [1.12, 1.92]	364	-0.216	0.662
Reproductive hormones					
AMH (ng/mL)	4.88 [4.69, 5.37]	6.01 [4.53, 10.3]	990	-0.516	0.516
LH (lU/L)	6.95 [4.56, 10.5]	7.21 [4.96, 10.4]	1	-0.002	0.655
FSH (lU/L)	5.85 [5.09, 6.73]	5.86 [5.01, 6.91]	1	0.086	0.951
Binary endpoints					
TG/HDL-C > 3.5, n (%)	4/84 (4.8%)	66/969 (6.8%)	2	-0.087	
Non-HDL-C ≥ 130 (mg/dL), n (%)	19/84 (22.6%)	205/969 (21.2%)	2	0.036	
OGTT 120-min glucose ≥ 140 (mg/dL), n (%)	14/79 (17.7%)	142/956 (14.9%)	20	0.056	
Euthyroid by TSH, n (%)	69/84 (82.1%)	937/970 (96.6%)	1	-0.465	

TSH, thyroid-stimulating hormone; Anti_TPO, anti-thyroid peroxidase antibodies; TG, triglycerides; HDL-C, high-density lipoprotein cholesterol; TC, total cholesterol; LDL-C, low-density lipoprotein cholesterol; glu0, fasting glucose; glu120, glucose concentration 120 minutes after oral glucose load; TT, total testosterone; FT, free testosterone; DHEAS, dehydroepiandrosterone sulphate; AMH, Anti-Müllerian hormone; LH, luteinizing hormone; FSH, follicle-stimulating hormone; SHBG, sex hormone-binding globulin.

**Figure 1:**
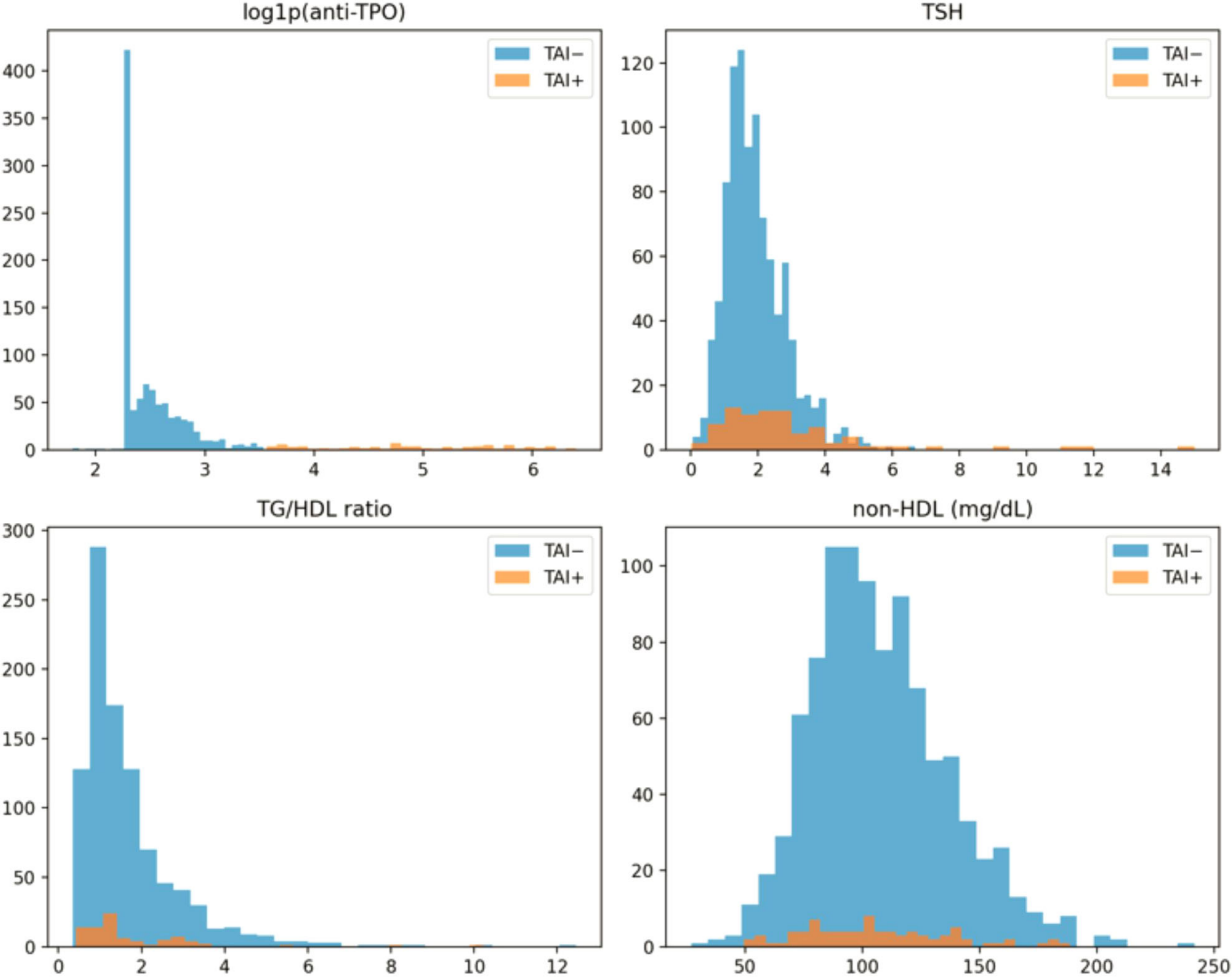
Distribution of thyroid and cardiometabolic.

Distributions of selected thyroid and cardiometabolic variables by TAI status are illustrated in **Figure 1**.

**Figure 2:**
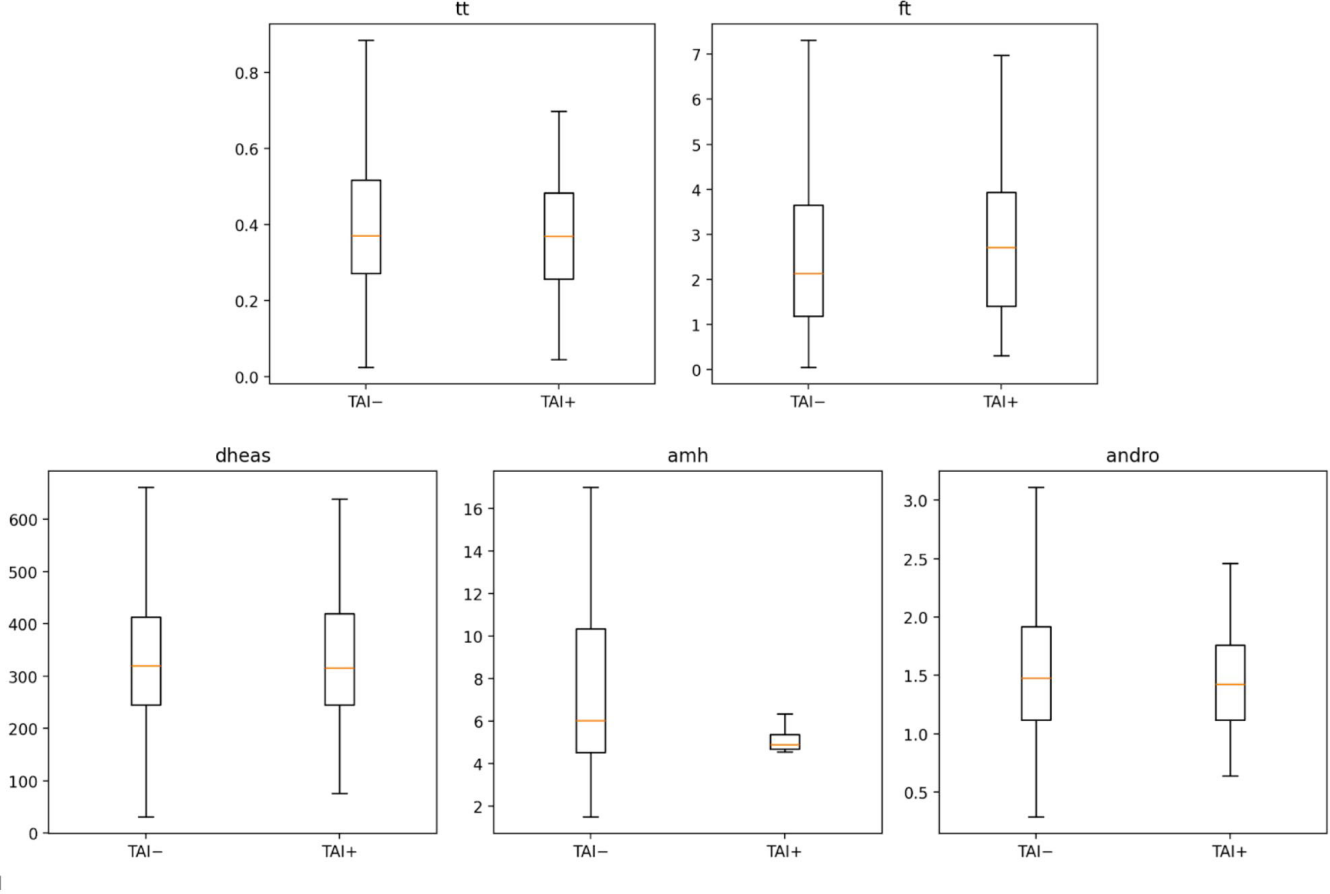
Androgen distributions.

In contrast, no clinically meaningful differences were observed between TAI-positive and TAI-negative participants for lipid parameters, glucose metabolism markers, or androgen-related variables, including TG, HDL-C, TG/HDL-C ratio, non-HDL-C, fasting glucose, or androgen indices (**Figure 2**). Exploratory comparisons of multiple biochemical and hormonal markers confirmed that TSH was the only variable remaining statistically significant after false discovery rate correction, whereas no androgenic or cardiometabolic parameters showed significant group differences.

**Figure 3:**
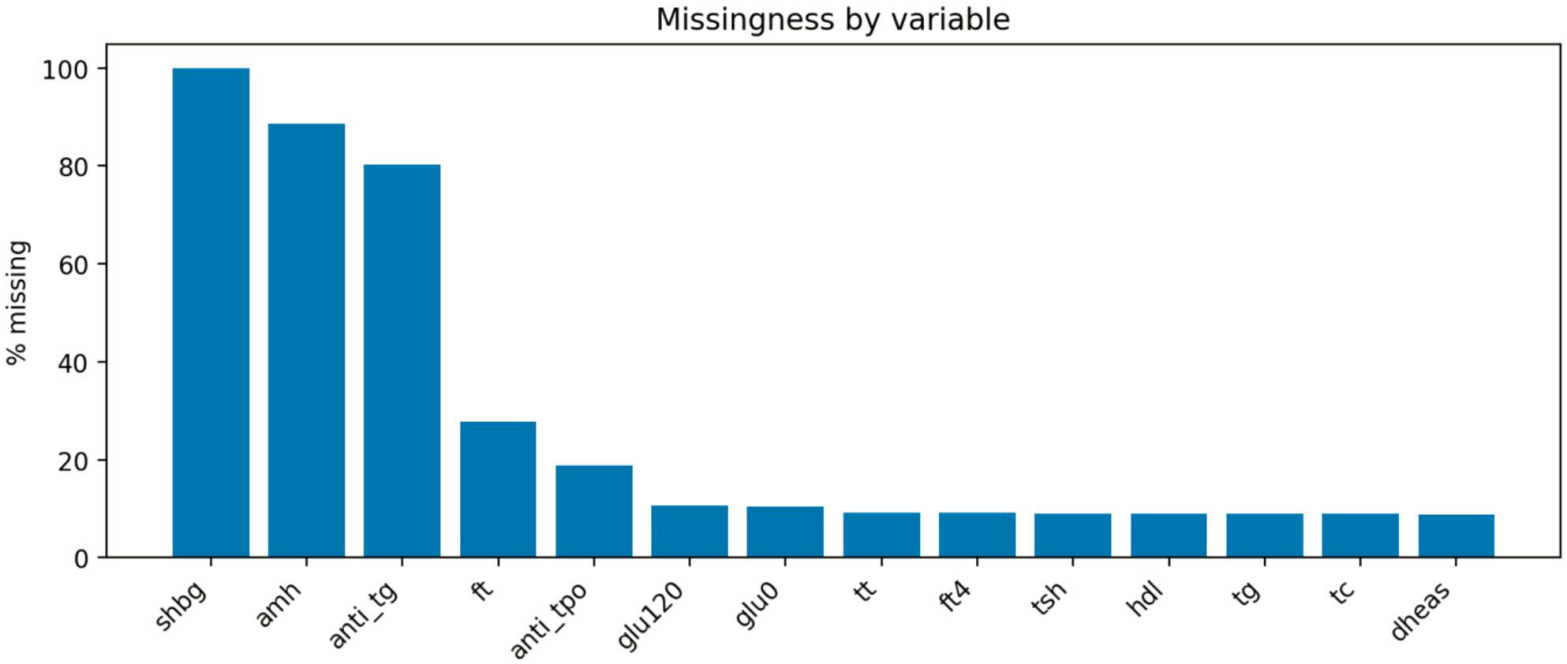
Missingness pattern. SHBG, sex hormone-binding globulin; amh, Anti-Müllerian hormone; anti_tg, anti-thyroglobulin antibodies; ft, free testosterone; anti_tpo, anti-thyroid peroxidase antibodies; glu120, glucose concentration 120 minutes after oral glucose load; glu0, fasting glucose; tt, total testosterone; fsh, follicle-stimulating hormone; ft4, free thyroxine; tsh, thyroid-stimulating hormone; hdl, high-density lipoprotein cholesterol; tg, triglycerides; tc, total cholesterol; dheas, dehydroepiandrosterone sulfate.

Patterns of missing data were evaluated descriptively. Anti-TPO measurements were available in 1,055 of 1,300 women (81.2%), while other cardiometabolic variables generally had substantially lower missingness. For example, TG, total cholesterol, HDL-C, TSH, and DHEAS had missingness rates below approximately 10%, whereas higher missingness was observed for selected reproductive hormones such as AMH and SHBG. The distribution of missing values across variables is shown in **Figure 3**.

Importantly, missingness patterns for key cardiometabolic variables were comparable between TAI-positive and TAI-negative groups, suggesting no major differential missingness that could bias exposure–outcome comparisons.

### 3.2 Primary endpoint analysis

The association between thyroid autoimmunity and the primary cardiometabolic endpoint was evaluated using age-adjusted Firth logistic regression in the complete-case analytic cohort.

Among 1,053 participants with available exposure and outcome data, the primary endpoint (TG/HDL-C ratio >3.5) was observed in 70 women. The event occurred in 4 of 84 TAI-positive participants and 66 of 969 TAI-negative participants.

In the age-adjusted model, thyroid autoimmunity defined by TAI_A was not associated with the primary endpoint. The estimated odds ratio was 0.77 (95% CI 0.21–1.67), indicating no statistically significant association between TAI status and elevated TG/HDL-C ratio.

Adjustment for age did not materially change the direction or magnitude of the effect estimate. The point estimate was below unity, but the confidence interval was wide and crossed 1.0, indicating substantial uncertainty and no evidence of a clinically meaningful increase in cardiometabolic risk associated with thyroid autoimmunity.

**Figure 4:**
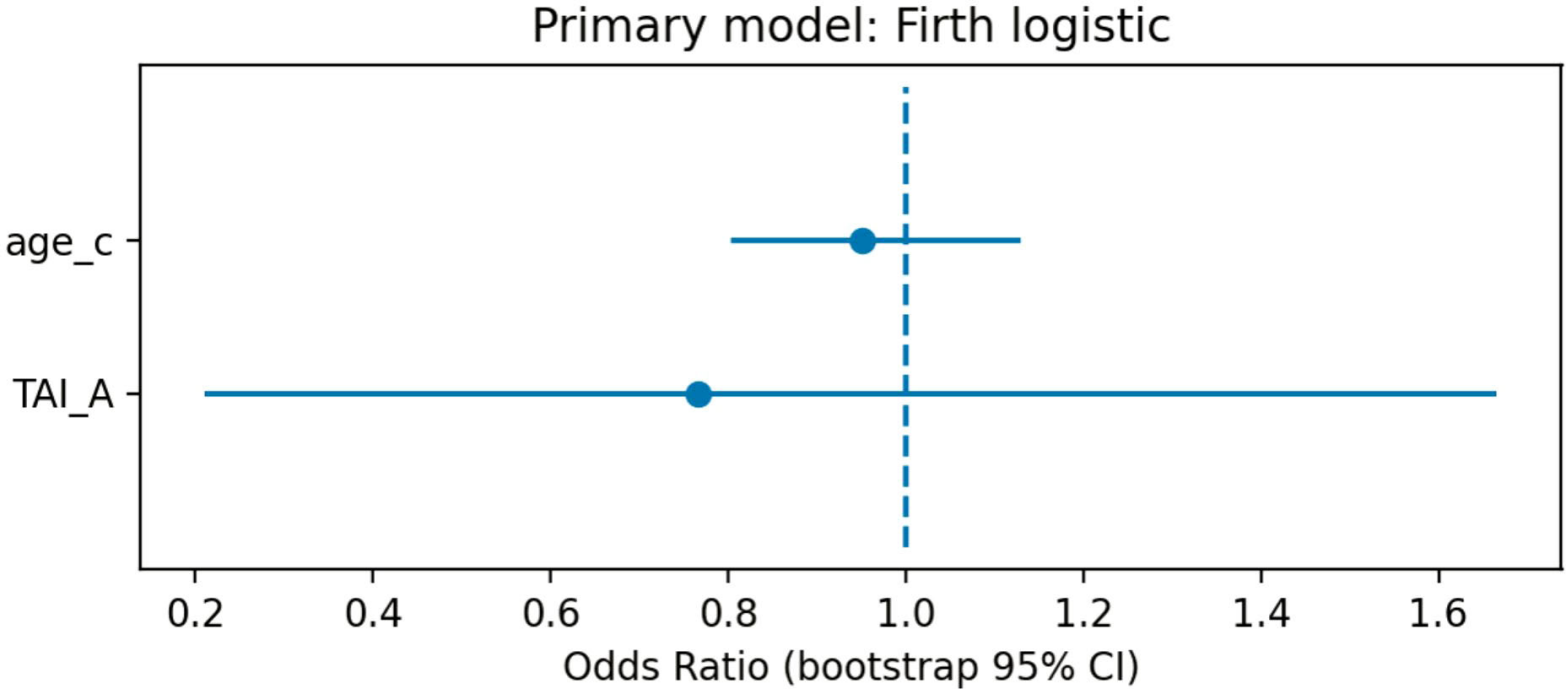
Forest plot of the age-adjusted Firth logistic regression model for the primary endpoint (TG/HDL-C ratio >3.5).

A forest plot summarizing the primary regression model is presented in **Figure 4**.

**Figure 5:**
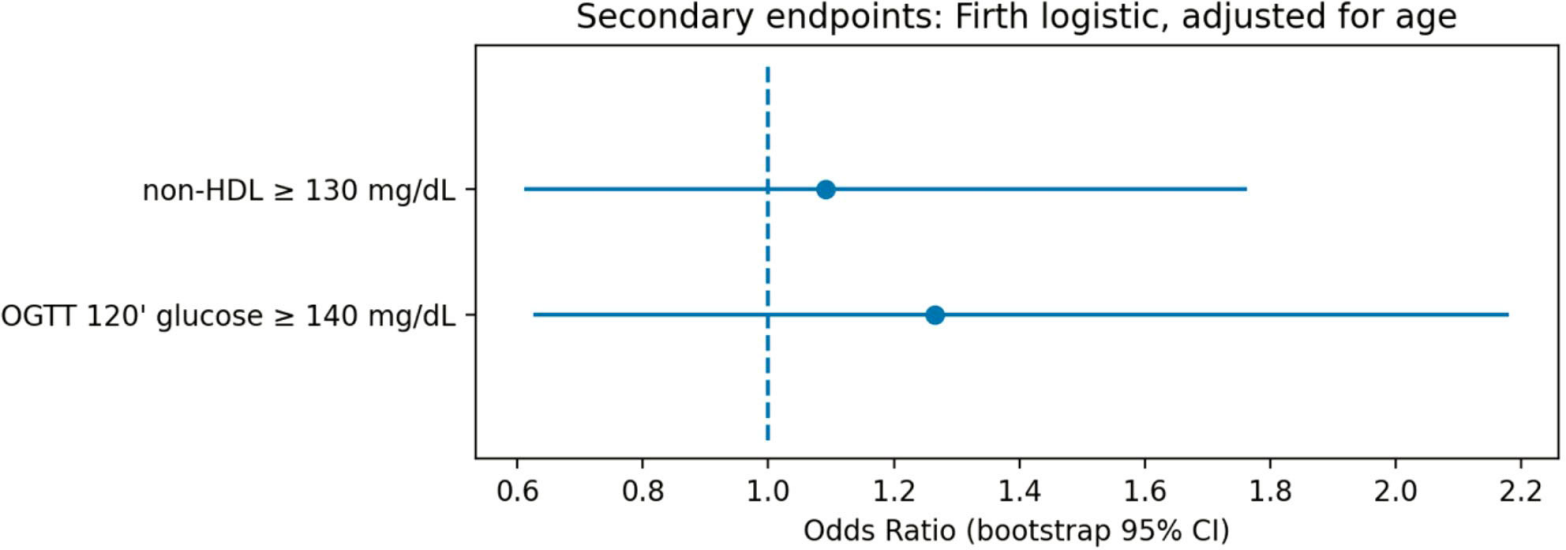
Calibration plot comparing predicted and observed probabilities for the primary endpoint (TG/HDL-C ratio >3.5).

Model calibration analysis demonstrated reasonable agreement between predicted and observed event probabilities, with no evidence of systematic miscalibration (**Figure 5**).

### 3.3 Secondary endpoints

Associations between thyroid autoimmunity and the pre-specified secondary cardiometabolic endpoints were evaluated using age-adjusted Firth logistic regression models.

For elevated non–HDL-C (≥130 mg/dL), the endpoint occurred in 19 of 84 TAI-positive participants and in 205 of 969 TAI-negative participants. In the age-adjusted model, thyroid autoimmunity defined by TAI_A was not significantly associated with elevated non–HDL-C (OR 1.09, 95% CI 0.61–1.76; n = 1053, events = 224).

For impaired glucose tolerance defined as 120-minute OGTT glucose ≥140 mg/dL, the endpoint was observed in 14 of 79 TAI-positive participants and in 142 of 956 TAI-negative participants. Age-adjusted regression analysis similarly did not demonstrate a significant association between TAI_A and impaired glucose tolerance (OR 1.27, 95% CI 0.63–2.18; n = 1035, events = 156).

Effect estimates for both secondary endpoints are summarized in **Figure 5**. Across both models, point estimates were close to the null value and the confidence intervals included unity, indicating no statistically significant associations between thyroid autoimmunity and these cardiometabolic outcomes.

### 3.4 Androgenic and hormonal group differences

Exploratory comparisons of continuous hormonal and metabolic parameters between TAI-positive and TAI-negative participants were conducted using nonparametric group comparisons with adjustment for multiple testing using the Benjamini–Hochberg false discovery rate (FDR).

A total of 16 candidate markers were evaluated in participants with defined thyroid autoimmunity status (TAI-positive n = 84; TAI-negative n = 971). Among these variables, thyroid-stimulating hormone (TSH) showed the largest standardized group difference and remained statistically significant after FDR correction (Hedges g = 0.90, FDR-adjusted p < 0.001), consistent with the expected alteration of thyroid axis parameters in individuals with thyroid autoimmunity.

In contrast, no androgen-related parameters—including total testosterone, free testosterone, DHEAS, or androstenedione—demonstrated statistically significant differences after FDR adjustment. Similarly, no lipid or glucose metabolism markers showed significant group differences.

**Figure 6:**
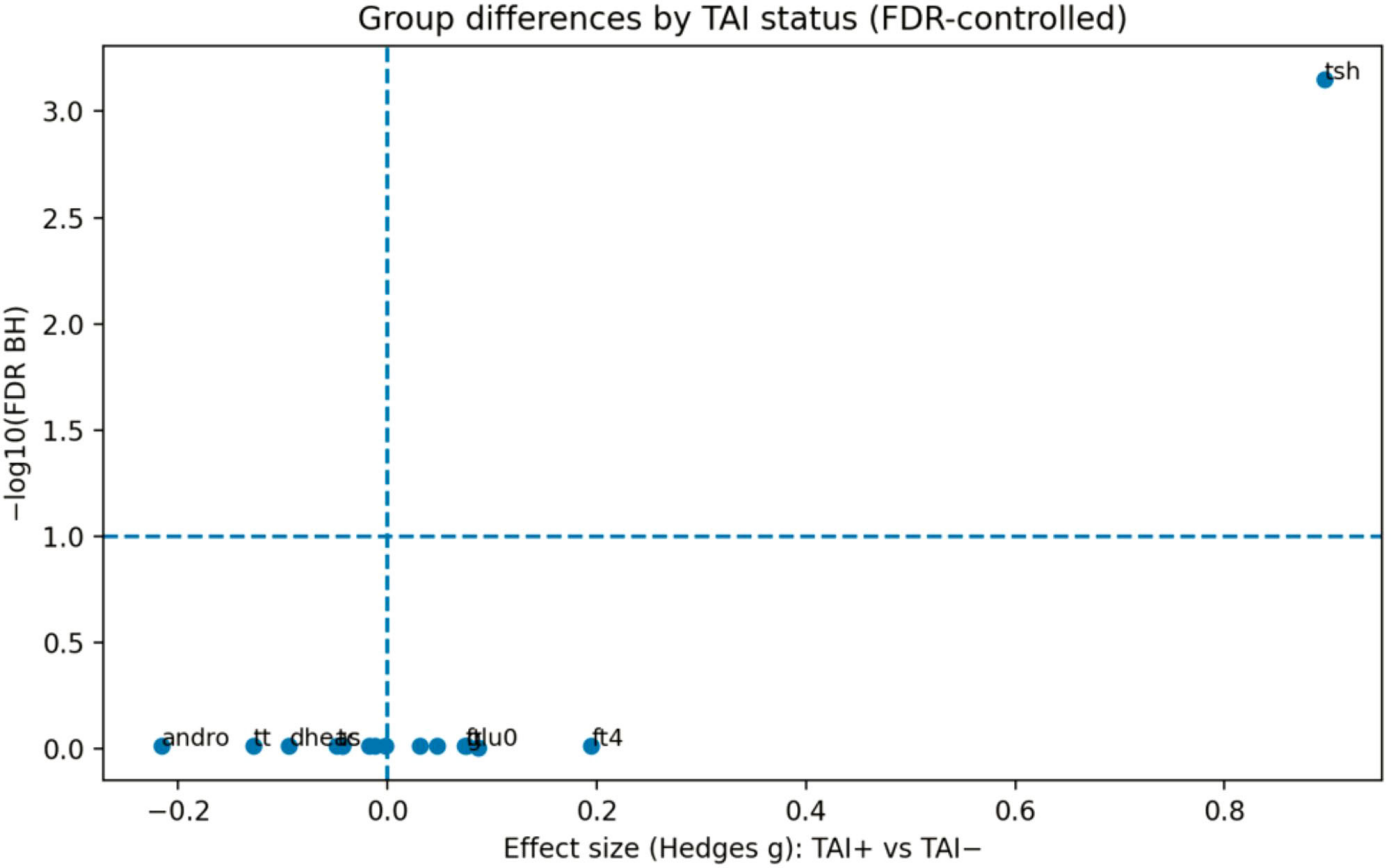
Volcano plot of hormonal and metabolic group differences between TAI-positive and TAI-negative women with PCOS.

**Figure 7:**
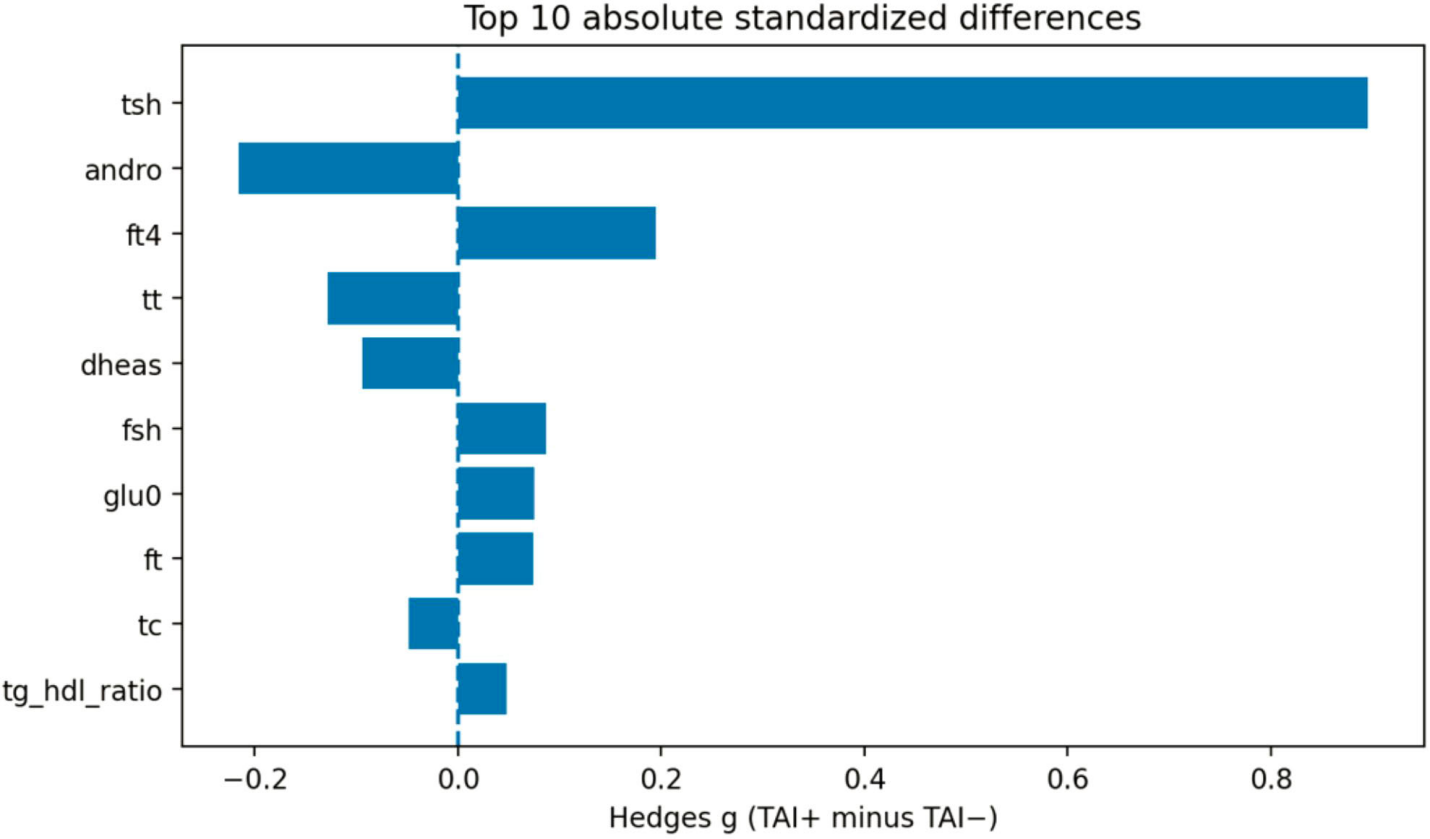
Ranked standardized effect sizes for hormonal and metabolic markers according to thyroid autoimmunity status. tsh, thyroid-stimulating hormone; andro, androstenedione; ft4, free thyroxine; tt, total testosterone; dheas, dehydroepiandrosterone sulfate; fsh, follicle-stimulating hormone; glu0, fasting glucose; ft, free testosterone; tc, total cholesterol; tg_hdl_ratio, triglycerides-to-high density lipoprotein cholesterol ratio.

These results indicate that, aside from the expected elevation of TSH in TAI-positive individuals, no consistent androgenic or cardiometabolic signal was detected across the evaluated markers. The distribution of effect sizes and adjusted significance levels is illustrated in the volcano plot (**Figure 6**) and the ranked effect size plot (**Figure 7**).

### 3.5 Sensitivity analyzes

The robustness of the primary findings was evaluated using several pre-specified sensitivity analyzes, including restriction to euthyroid participants, alternative definitions of thyroid autoimmunity, and trimming of extreme values.

When restricting the analysis to euthyroid participants, the association between TAI_A and the primary endpoint remained non-significant (OR 0.50, 95% CI 0.09 - 1.28; n = 1005). Similarly, alternative exposure definitions based on anti-TPO positivity combined with elevated TSH (TAI_B) and high-titer anti-TPO positivity (TAI_C) did not yield statistically significant associations with the primary endpoint (TAI_B: OR 2.87, 95% CI 0.47 - 8.77; TAI_C: OR 1.28, 95% CI 0.34 - 2.90).

Trimming extreme values at the 1st and 99th percentiles also did not materially alter effect estimates for the primary model (OR 0.49, 95% CI 0.08 - 1.20; n = 1033).

**Figure 8:**
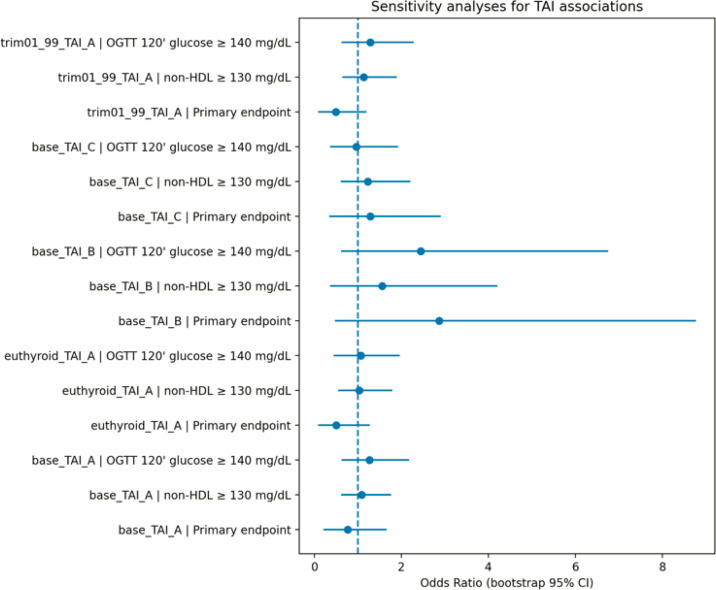
Robustness analysis of the association between thyroid autoimmunity and the primary cardiometabolic endpoint across sensitivity models.

Across these analytic scenarios, confidence intervals remained wide and consistently included the null value. Effect estimates across all sensitivity models are summarized in the robustness forest plot (**Figure 8**).

### 3.6 Model diagnostics

Model diagnostics supported appropriate specification of the regression models.

Because the primary analyzes were performed using Firth logistic regression, selected diagnostic procedures not directly available for Firth models were evaluated using auxiliary standard logistic models fitted to the same complete-case cohorts.

Calibration plots indicated adequate agreement between predicted and observed risks for the primary and secondary endpoints. Hosmer–Lemeshow goodness-of-fit tests did not indicate evidence of poor calibration in these auxiliary models.

Multicollinearity among predictors was minimal, with variance inflation factors remaining low. Evaluation of influential observations using Cook’s distance did not identify individual observations exerting disproportionate influence on model estimates.

Sensitivity analyzes using spline-based modeling of the age covariate produced results consistent with the primary models assuming linear age effects. Detailed diagnostic results are provided in **Supplementary Tables 1**, **S2** and **Supplementary Figures 1**, **S2**.

## 4 Discussion

Thyroid autoimmunity has been frequently observed in women with PCOS (18), and several studies have suggested potential associations with adverse metabolic characteristics, including dyslipidemia, insulin resistance, or impaired glucose tolerance (5, 6, 19, 20). Potential mechanisms linking PCOS and thyroid autoimmunity include shared pathways such as chronic low-grade inflammation, immune dysregulation, and genetic susceptibility; however, these mechanisms do not appear to directly translate into adverse metabolic phenotypes (7, 9). Recent studies (2024–2025) further support increased autoimmune activity in PCOS, while continuing to report heterogeneous and sometimes conflicting associations with metabolic outcomes (7, 21).

In our pre-specified cross-sectional analysis of a large cohort of women with PCOS, thyroid autoimmunity, defined using several *a priori* criteria, was not associated with a more adverse cardiometabolic or androgenic profile. No significant association was observed between thyroid autoimmunity and the primary endpoint (TG/HDL-C >3.5), nor with secondary endpoints including non-HDL-C ≥130 mg/dL and 120-minute OGTT glucose ≥140 mg/dL. Effect estimates were modest, confidence intervals included the null value, and findings remained consistent across multiple sensitivity analyzes, including alternative definitions of thyroid autoimmunity, restriction to euthyroid participants, and trimming of extreme values. Exploratory comparisons of continuous hormonal and metabolic parameters, adjusted for false discovery rate, did not identify meaningful differences between TAI-positive and TAI-negative women. The only differences observed concerned thyroid axis parameters, most notably higher TSH levels in individuals with thyroid autoimmunity. Our results are consistent with reports that did not confirm such relationships. For example, Gawron et al. (22) found no significant association between PCOS phenotypes and thyroid dysfunction or anti-thyroid antibodies. Similarly, Skrzyńska et al. (23) reported no relationship between thyroid abnormalities and key hormonal parameters. Together with our findings, these data suggest that thyroid autoimmunity alone may not be a major determinant of metabolic or androgenic severity in PCOS.

However, studies by Romitti et al., Suchta et al., and Ohikhuai et al. have suggested that thyroid autoimmunity may be associated with metabolic disturbances contributing to increased cardiovascular risk in women with PCOS (6, 7, 24).

Several factors may explain discrepancies between studies. First, many previous analyzes included participants with mixed thyroid functional states or did not specifically evaluate euthyroid subgroups. Given the well-established metabolic effects of thyroid hormones, even subtle thyroid dysfunction could confound observed associations. In the present study, restriction to euthyroid participants did not materially alter the results, arguing against a major role of thyroid function variability in explaining the lack of association.

Second, heterogeneity in the definition of thyroid autoimmunity may contribute to inconsistent findings. Previous studies have used different antibody thresholds or binary classifications without considering antibody titers. By contrast, the present study applied multiple pre-specified definitions, including a high-titer anti-TPO threshold, providing a more comprehensive characterization of thyroid autoimmunity. None of these approaches yielded consistent associations with cardiometabolic endpoints, supporting the robustness of the observed null findings.

Third, differences in sample size and statistical methodology may also play a role. Smaller studies are more susceptible to residual confounding and type I error, particularly when multiple outcomes are assessed. The present analysis, conducted in a relatively large cohort and incorporating bootstrap-based confidence intervals as well as correction for multiple comparisons, reduces the likelihood that the observed findings are attributable to statistical instability.

From a pathophysiological perspective, although thyroid autoimmunity reflects immune dysregulation and may share common mechanisms with PCOS, such as chronic low-grade inflammation or altered immune-endocrine interactions, it does not appear to translate into a clinically meaningful worsening of cardiometabolic or androgenic features. It is plausible that previously reported associations were mediated primarily through thyroid dysfunction rather than autoimmunity per se. From a clinical perspective, these findings help refine the interpretation of thyroid autoimmunity in women with PCOS. Although thyroid autoimmunity and PCOS frequently co-occur, the presence of anti-TPO antibodies alone does not appear to identify a subgroup at increased cardiometabolic risk. While screening for thyroid dysfunction remains clinically appropriate, the present results do not support the use of thyroid autoimmunity as an independent marker of metabolic severity in young women with PCOS.

At the same time, these conclusions should be interpreted with caution. The cross-sectional design precluded causal inference, and the relatively young age of the study population limits the assessment of long-term outcomes. Further prospective studies are needed to determine whether thyroid autoimmunity may influence cardiometabolic trajectories over time or contribute to risk in specific subgroups of patients with PCOS.

Taken together, these results suggest that while thyroid autoimmunity is biologically relevant with respect to thyroid axis alterations, it does not appear to independently delineate a metabolically or androgenically more severe PCOS phenotype in this cohort of women with PCOS.

### 4.1 Strengths

This study has several methodological strengths.

First, the exposure definitions and cardiometabolic endpoints were specified *a priori*, which reduces the risk of *post hoc* hypothesis generation and improves interpretability of the findings.

Second, thyroid autoimmunity was evaluated using multiple pre-specified operational definitions, including a stringent high-titer anti-TPO threshold. This approach allowed the robustness of the results to be assessed across alternative definitions of thyroid autoimmunity.

Third, the analysis incorporated several sensitivity strategies, including restriction to euthyroid participants, trimming of extreme values, and bootstrap-based confidence interval estimation. Across these analytic scenarios, the results remained consistent and did not indicate a stable association between thyroid autoimmunity and cardiometabolic endpoints.

Fourth, exploratory hormonal and metabolic comparisons were evaluated using correction for multiple testing with the Benjamini–Hochberg false discovery rate procedure, reducing the risk of false-positive findings.

Finally, the analytical workflow was fully reproducible and implemented using version-controlled scripts with clearly documented preprocessing rules, model diagnostics, and computational environment, which enhances transparency and reproducibility of the study.

### 4.2 Limitations

Several limitations of this study should be acknowledged.

First, the cross-sectional design precludes causal inference and does not allow assessment of longitudinal cardiometabolic outcomes. The present analysis therefore cannot determine whether thyroid autoimmunity influences long-term metabolic trajectories in women with PCOS.

Second, body mass index (BMI) was not available in the dataset and could not be included in the regression models. Given the central role of adiposity in both PCOS pathophysiology and cardiometabolic risk, residual confounding related to obesity cannot be excluded.

Third, information on thyroid hormone replacement therapy, including levothyroxine treatment, was not available in the dataset. As a result, sensitivity analyzes excluding participants receiving thyroid hormone therapy could not be performed. Because individuals treated with levothyroxine may differ metabolically from untreated individuals with thyroid autoimmunity, the absence of treatment data represents an additional source of potential confounding.

Fourth, some androgen-related parameters were not available with sufficient completeness to construct composite indices such as the free androgen index (e.g., due to missing SHBG measurements). This may limit the granularity of androgenic phenotype characterization in the present cohort.

Fifth, the number of events for the primary endpoint was relatively small in the TAI-positive group, which contributes to wide confidence intervals and limited precision of effect estimates despite the use of Firth logistic regression.

Finally, the study population consisted of women evaluated in a single clinical setting and primarily represented relatively young patients with PCOS. These characteristics may limit the generalizability of the findings to other populations or to older women with PCOS.

## 5 Conclusion

In this pre-specified cross-sectional analysis of women with PCOS, thyroid autoimmunity was not associated with an adverse cardiometabolic or androgenic phenotype. Across multiple operational definitions of thyroid autoimmunity and several sensitivity analyzes, no consistent associations were observed with elevated TG/HDL-C ratio, non-HDL-C, impaired glucose tolerance, or androgen-related parameters.

Although thyroid autoimmunity was associated with expected alterations in thyroid axis markers, it did not delineate a subgroup of women with PCOS characterized by greater metabolic or androgenic severity under the studied conditions. These findings suggest that anti-TPO positivity alone should not necessarily be interpreted as an indicator of increased cardiometabolic risk in young women with PCOS.

Further prospective studies incorporating longitudinal follow-up and comprehensive metabolic profiling are needed to clarify whether thyroid autoimmunity influences long-term cardiometabolic outcomes in this population.

## Data Availability

The datasets presented in this study can be found in online repositories. The names of the repository/repositories and accession number(s) can be found in the article/**Supplementary Material**.

## Supplementary material

Supplementary Figure 1 Calibration plot for the primary regression model.

Supplementary Figure 2 Influence diagnostics for the primary regression model.

Supplementary Table 1 Hosmer–Lemeshow goodness-of-fit tests for auxiliary logistic regression models used in model diagnostics.

Supplementary Table 2 Variance inflation factors (VIF) for predictors included in regression models.
