# Supplementary figures and images for: Thyroid autoimmunity does not delineate a cardiometabolic or androgenic phenotype in women with polycystic ovary syndrome: a pre-specified cross-sectional analysis
Source: Front Endocrinol (Lausanne). 2026 May 1;17:1839476. doi: 10.3389/fendo.2026.1839476 (PMC13175869; doi:10.3389/fendo.2026.1839476)

**Supplementary Figure S1.** Calibration plot for the primary regression model.


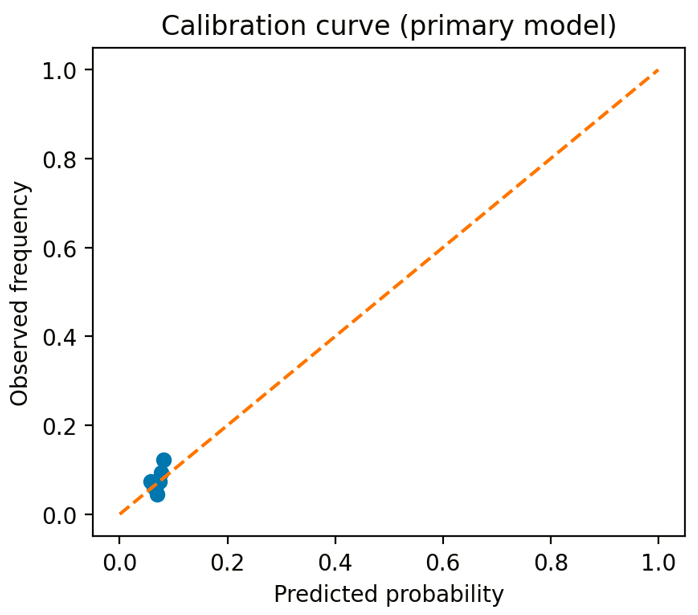

Supplement: Supplementary Figure 1 — Calibration plot for the primary regression model. [file DataSheet1.docx]

**Supplementary Figure S2**. Influence diagnostics for the primary regression model.


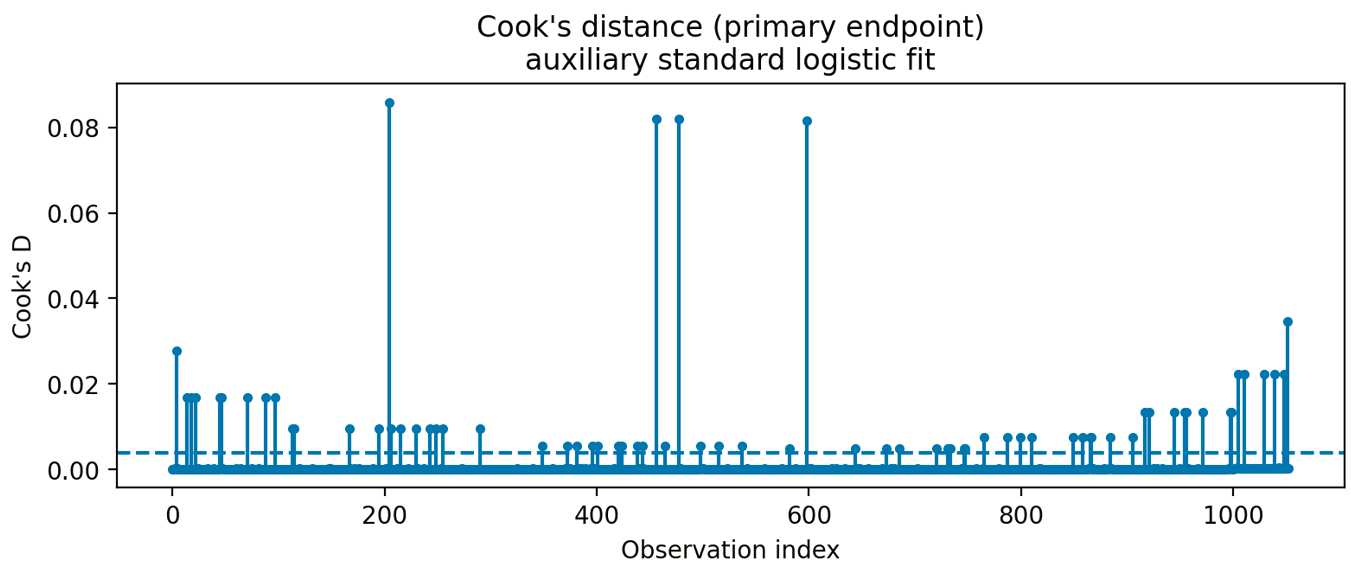

Supplement: Supplementary Figure 2 — Influence diagnostics for the primary regression model. [file DataSheet2.docx]
