# Supplementary material for: Thyroid autoimmunity does not delineate a cardiometabolic or androgenic phenotype in women with polycystic ovary syndrome: a pre-specified cross-sectional analysis
Source: Front Endocrinol (Lausanne). 2026 May 1;17:1839476. doi: 10.3389/fendo.2026.1839476 (PMC13175869; doi:10.3389/fendo.2026.1839476)
Supplement: Supplementary Table 1 — Hosmer–Lemeshow goodness-of-fit tests for auxiliary logistic regression models used in model diagnostics. [file Table1.docx]

**Supplementary Table S1.** Hosmer–Lemeshow goodness-of-fit tests for auxiliary logistic regression models used in model diagnostics.

| **Model set** | **Endpoint** | **Note** | **hl stat** | **hl df** | **hl p** | **hl groups used** |
| --- | --- | --- | --- | --- | --- | --- |
| primary | ep_primary | Descriptive only; interpret cautiously, especially with sparse events. | 12.432 | 8.000 | 0.133 | 10.000 |
| secondary_non_hdl | ep_non_hdl | Descriptive only; interpret cautiously, especially with sparse events. | 15.777 | 8.000 | 0.046 | 10.000 |
| secondary_ogtt120 | ep_ogtt120 | Descriptive only; interpret cautiously, especially with sparse events. | 2.916 | 8.000 | 0.940 | 10.000 |
