# Supplementary material for: Thyroid autoimmunity does not delineate a cardiometabolic or androgenic phenotype in women with polycystic ovary syndrome: a pre-specified cross-sectional analysis
Source: Front Endocrinol (Lausanne). 2026 May 1;17:1839476. doi: 10.3389/fendo.2026.1839476 (PMC13175869; doi:10.3389/fendo.2026.1839476)
Supplement: Supplementary Table 2 — Variance inflation factors (VIF) for predictors included in regression models. [file Table2.docx]

**Supplementary Table S2.** Variance inflation factors (VIF) for predictors included in regression models.

| term | VIF | model set | endpoint |
| --- | --- | --- | --- |
| TAI | 1.087 | primary | ep_primary |
| Age | 1.087 | primary | ep_primary |
| TAI | 1.087 | secondary_non_hdl | ep_non_hdl |
| Age | 1.087 | secondary_non_hdl | ep_non_hdl |
| TAI | 1.083 | secondary_ogtt120 | ep_ogtt120 |
| Age | 1.083 | secondary_ogtt120 | ep_ogtt120 |
